# Editorial Note: The excitotoxin quinolinic acid induces tau phosphorylation in human neurons

**DOI:** 10.1371/journal.pone.0323774

**Published:** 2025-05-08

**Authors:** The PLOS One Editors

This Editorial Note is issued to provide an update to the Expression of Concern notice previously issued on this article [1,2].

After the Expression of Concern notice [2] was issued, the authors responded to the concerns raised with the results presented in Figs 4 and 8 [1,2]. The first author stated that the Tau panels in Figs 4A–4C represent the same experimental results, and that the data in the graphs in Fig 4 are based on five separate experiments, while the representative blots with each graph are from a single experiment. They stated that four membranes were prepared, one was blotted with total tau, and the other three with the phospho-tau antibodies AT8, AT180 and AT270. They stated that the same band of total tau was used for normalizing the signal for the three phospho-tau antibodies (Figs 4A, B, and C), and a different total tau band was used for the PHF antibody (Fig 4D) as the amount of protein loaded on the gel for PHF (10 μg) was different than that loaded for the other three antibodies (20 μg). A member of the *PLOS One* Editorial Board reviewed this response and stated that the explanation that the control for Figs 4A-C is the same is reasonable because it represents the same set of replicates from the same biological replicate. The first author stated that the original western blots underlying Fig 4 are no longer available but the underlying data for the graphs in Fig 4 are provided here in S1-S2 Files.

Regarding the AT8 blot in Fig 8A, the first author stated that while running the gel for this blot, the sample in lane 4 was loaded twice in two adjacent lanes, and in order to align this blot with the lanes in the blot for Tau, lane 5 was excised out and the other lanes shifted to match with the lanes for Tau. The underlying AT8 blot in Fig 8A is provided here in S3 File. The first author stated that the underlying Tau blot in Fig 8A and the underlying Tau and AT180 blots in Fig 8B are no longer available.

The *PLOS One* Editors remain concerned about the described practice of normalizing samples against a control obtained from a separate blot, and recommend that the quantified western blot results presented in this article [1] are interpreted with caution.

During editorial review of the author’s response following the publication of [2], a member of the *PLOS One* Editorial Board noted that fluorescence appears saturated for the blue channel in Fig 3, panel B middle image (Tau 180), and that the authors’ explanation that the DAPI stained results were not used for quantification did not resolve their concern. The corresponding author stated that underlying images for Figs 3A-C are no longer available. In the absence of the individual-level data underlying the graph and the original image data used for the quantification of the results presented in Fig 3, the validity of the quantification results cannot be verified and these results should be interpreted with caution.

The authors stated that with the exception of S1-S3 Files, the underlying data for this article [1,2] are no longer available.

Based on our assessment, the explanations and data provided by the authors provide some clarification regarding the image duplications and splice line concerns reported in [2], but the authors’ explanation raises additional concerns about the reliability of the quantified western blot results presented in this article. Therefore, the *PLOS One* Editors issue this Editorial Note to provide an update to the published Expression of Concern [2], to share the available underlying data and outcome of the editorial assessment of these data, and to inform readers of the additional concern raised with Fig 3.

## Supporting information

S1 File Underlying data supporting the graphs in Figs 4A-C. (XLS)

S2 File Underlying data supporting the graphs in Figs 4A-D. (XLS)

S3 File Cropped underlying blot for the AT8 panel in Fig 8A. (XLS)
