# Supplementary figures and images for: Editorial Note: The excitotoxin quinolinic acid induces tau phosphorylation in human neurons
Source: PLoS One. 2025 May 8;20(5):e0323774. doi: 10.1371/journal.pone.0323774 (PMC12061166; doi:10.1371/journal.pone.0323774)

Original AT8 blot for Figure 8A

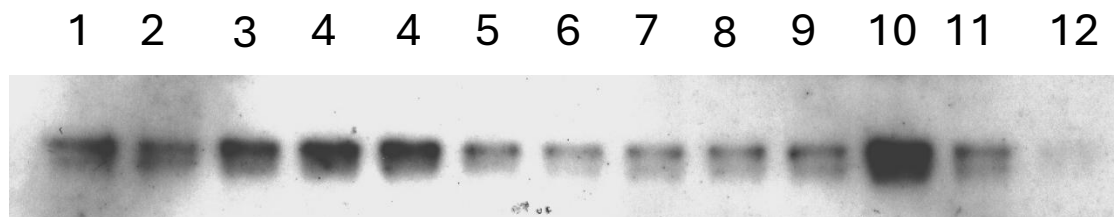

Supplement: S3 File — (XLS) [file pone.0323774.s003.pdf]
